# Protein identification and potential bioactive peptides from pumpkin (*Cucurbita maxima*) seeds

**DOI:** 10.1002/fsn3.4188

**Published:** 2024-04-24

**Authors:** Chu‐Ti Lin, Lhumen A. Tejano, Fenny Crista A. Panjaitan, Vinny Nabila Surya Permata, Tesalonika Sevi, Yu‐Wei Chang

**Affiliations:** ^1^ Department of Food Science National Taiwan Ocean University Keelung Taiwan; ^2^ Institute of Fish Processing Technology, College of Fisheries and Ocean Sciences University of the Philippines Visayas Miagao Iloilo Philippines; ^3^ Marine Products Processing Study Program Marine and Fisheries Polytechnic of Jembrana Bali Indonesia

**Keywords:** bioactive peptide, enzymatic hydrolysis, proteomics, pumpkin seed

## Abstract

Pumpkin is an economically important crop all over the world. Approximately, 18%–21% of pumpkins, consisting of peels and seeds by‐products, are wasted during processing. In addition, the seeds are rich in protein and have the potency of bioactive peptide production. This study aims to recognize the proteins and investigate the potential bioactive peptides from pumpkin (*Cucurbita maxima*) seeds. Pumpkin seeds were subjected to hot air drying (HAD) at 55°C for 12 h and freeze‐drying (FD) at −80°C for 54 h before they were powdered, analyzed, and precipitated by isoelectric point to obtain pumpkin seed protein isolates (PSPI). PSPI comprised 11S globulin subunit beta, 2S seed storage albumin, and chaperonin CPN60‐1. To generate hydrolysate peptides, PSPI was hydrolyzed using papain, pepsin, and bromelain. FD group pepsin hydrolysates had the highest peptide content of 420.83 mg/g. ACE inhibition and DPP‐IV inhibition activity were analyzed for each enzymatic hydrolysate. The pepsin hydrolyzed sample exhibited the highest ACE inhibition of 70.26%, and the papain hydrolyzed sample exhibited the highest DPP‐IV inhibition of 30.51%. The simulated gastrointestinal digestion (SGID) conducted by pepsin and pancreatin increased ACE inhibitory activity from 76.93% to 78.34%, and DPP‐IV inhibited activity increased from 58.62% to 77.13%. Pepsin and papain hydrolysates were fractionated using ultrafiltration to measure ACE and DPP‐IV inhibition activity. The highest free radical scavenging abilities were exhibited by the <1 kDa hydrolysate fractions with 78.34% ACE inhibitory activities and 79.55% DPP‐IV inhibitory activities. This research revealed that pumpkin seeds had the potency to produce bioactive peptides.

## Graphical abstract

Pumpkin seeds protein isolates comprised 11S globulin subunit beta, 2S storage albumin, and chaperonin CPN60‐1. Pepsin and papain protein hydrolysates showed ACE and DPP‐IV inhibition activities. The hydrolysate fractions (< 1 kDa) gave the highest ACE and DPP‐IV inhibition activities.

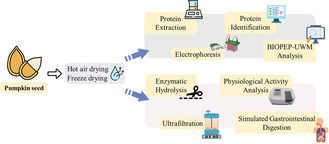

## 1 INTRODUCTION

Pumpkin belongs to *Cucurbitaceae Cucurbita* and is one of the significant economic crops worldwide. As stated by the Food and Agriculture Organization of the United Nations (FAO, 2021), the global pumpkin planting area exceeds 2 million hectares, with a total output of 27 million metric tons, and this production is continuously increasing each year. Apart from being consumed directly, pumpkins are commonly used as raw materials in various food products, such as pastries, jams, and soups. During the pumpkin processing, mostly only the flesh of the pumpkin is utilized, generating approximately 18% to 21% of by‐products, mainly peel and seeds (Nor et al., 2013). These have been widely used as animal feed, bakery, and raw material for snack food (Bombardelli & Morazzoni, 1997). However, pumpkin seeds are rich in protein (Quanhong & Caili, 2005), which can be great precursors of bioactive peptides, minerals (Amin et al., 2019), unsaturated fatty acids (Meru et al., 2018), phytoestrogens, and vitamins (Lestari & Meiyanto, 2018), making them worthy of research and maximum utilization.

Bioactive peptides typically comprise 2 to 20 amino acids with diverse functional groups, and their relatively small molecular size allows for rapid digestion and absorption by the human body (Xu et al., 2018). Bioactive peptides can be gained through enzymatic hydrolysis, autolysis, and microbial fermentation, and they have various physiological activities based on specific amino acid sequences. Since chemically synthesized drugs have various side effects, it is preferable to isolate and recognize bioactive peptides from natural food proteins that have the potential to be used as alternatives (Tu et al., 2018). Since the application of animal protein is limited in terms of environmental protection, the use of plant protein has been widely increased (Zivanovic et al., 2011). Various studies reported that the peptides produced by enzymatic hydrolysis of pumpkin seed protein have antioxidant (Amin et al., 2018), anti‐diabetes (Bayat et al., 2014), hypoglycemic (Kushawaha et al., 2017), anticancer (Jayaprakasam et al., 2003), antibacterial (Dissanayake et al., 2018), and other physiological activities, which show its prodigious potential in the manufacture of nutraceutical or pharmaceutical products.

This research aims to identify the proteins and investigate the potential bioactive peptides from pumpkin (*Cucurbita maxima*) seeds. The study used proteomic technology to identify its protein sequence, determine the physiological activity of the peptide released by enzymatic hydrolysis, and analyze its influence on human enzyme decomposition and molecular weight (MW) through simulated gastrointestinal digestion and ultrafiltration. For further application, the evaluation of pumpkin seed potential bioactive peptides can increase the value of pumpkin seeds.

## 2 METHODS AND MATERIALS

### 2.1 Materials

Pumpkin seeds (*Cucurbita maxima*) were provided by Xin Yue restaurant (New Taipei City, Taiwan). The seeds were cleaned with tap water to remove residual pulp and subsequently air‐dried for 12 h. Then, the seeds were subjected to heat drying at 55°C for 12 h and freeze‐drying at −80°C for 54 h. Dried pumpkin seed samples were homogenized into powder and then stored at −20°C for later use. Analytical‐grade chemicals are used for further analysis.

### 2.2 Protein content determination

The crude protein content of pumpkin seeds was analyzed according to the macro‐Kjeldahl method described in the Association of Analytical Chemists International (AOAC) (2005) handbook.

### 2.3 Pumpkin seed protein isolation

Pumpkin seed powder defatting was performed by Rodríguez‐Miranda et al. (2012) with minor modifications. The pumpkin seed powder was diluted with hexane at a 1:20 (w/v) ratio. Lipid extraction of the mixture was conducted at room temperature for 1.5 h, and the extraction process was repeated twice. After the extraction, the mixture was filtered by vacuum filtration to remove hexane. The pumpkin seed powder was dried using hot air at 55°C for 4 h to remove residual hexane. According to Fan et al. (2012), protein extraction was conducted with some modifications. The defatted pumpkin seed powder was diluted with water at a ratio of 1:30 (w/v). The pH sub‐sampler was adjusted to pH 11 using 1 N sodium hydroxide (NaOH) and stirred at 50°C for 1.5 h. The mixture was then centrifuged at 9,600 rpm for 30 min. The supernatant was recovered, and its pH was adjusted to 5.3 using 1 N hydrochloric acid (HCl) and centrifuged at 9,600 rpm for 30 min. The supernatant was removed, and the precipitate was recovered and freeze‐dried. This freeze‐dried product and the isolated pumpkin seed protein were stored at −20°C for further use. The total weight of pumpkin seed powder for protein extraction is classified as W_1_ (g). After the sample was extracted and freeze‐dried, the weight was classified as W_2_ (g). The extraction yield was designed using the formula below:

$$\text{Extraction yield of pumpkin seed protein isolate}\;\left(\%\right)=W_{2}/W_{1}\times 100\%.$$

### 2.4 Sodium dodecyl sulfate‐polyacrylamide gel electrophoresis

Gel for Sodium dodecyl sulfate‐polyacrylamide gel electrophoresis (SDS‐PAGE) determination was prepared according to Huang et al. (2015) and Laemmli (1970) with a 12% separating gel and a 4% stacking gel. A total 0.1 g pumpkin seed protein isolate was diluted with 1 mL sample buffer (ddH_2_O 3.55 mL, 0.5 M pH 6.8 Tris–HCl 1.25 mL, Glycerol 2.5 mL, 10% SDS 2 mL, 0.5% Bromophenol blue 0.2 mL, β‐Mercaptoethanol 0.05 mL), heated in a dry bath at 95°C for 5 min, centrifuged at 5,000 rpm for 5 min. The supernatant was recovered as the analysis sample solution. The 10× Running Buffer (Tris base 3.03 g, Glycine 14.4 g, SDS 1.0 g) was added into the electrophoresis chamber; 5 μL of protein ladder and 10 μL of sample analysis solution were injected into the wells on the gel. The power supply voltage was set to 70 V and then 120 V for stacking gel and resolving gel, respectively. The gel was then submerged in a staining solution (Coomassie Brilliant Blue R‐250 1.5 g, Methanol 500 mL, Acetic acid 100 mL, ddH_2_O 500 mL) and then shaken gently for 30 min. A destaining solution (H_2_O/Methanol/Acetic acid, 7:2:1, v/v/v) was used for destaining the gel until the background of the gel became transparent.

### 2.5 Proteomic approach and mascot database comparison

#### 2.5.1 Gel cutting and in‐gel digestion

The proteomics approach was performed by the method reported by Shevchenko et al. (2006) with slight modifications. The excised bands of SDS‐PAGE gel were divided into gel particles with dimensions of approximately 1 mm^3^ each and then placed into siliconized microcentrifuge tubes. A total of 200 μL of 25% acetonitrile/25 mM ammonium bicarbonate (NH_4_HCO_3_) was inserted into a microcentrifuge and repeated until the colloidal particles became transparent. Freshly prepared 50 mM dithioerythritol/25 mM NH_4_HCO_3_ for 100 μL was added and reacted at 37°C for 1 h to cut the disulfide bonds, and then the solution was discarded. Alkylation was performed by adding 100 μL of 100 mM iodoacetamide/25 mM NH_4_HCO_3_ at room temperature in the dark for 1 h and centrifuged to remove the solution. Drying was conducted by adding 200 μL 25% acetonitrile/25 mM NH_4_HCO_3_, reacted at room temperature for 5 min, centrifuged to remove the solution, then added with 100 μL 100% acetonitrile, reacted for 5 min at room temperature, and centrifuged to remove the solution. The concentrate was centrifuged to dry for 5 min. Then, the solution was drained into the microcentrifuge tube. This step was repeated 1–3 times until the colloidal particles became inelastic. Lys‐C protease solution (Enzyme: Sample = 1:50) and 25 mM NH_4_HCO_3_ were added until the liquid surface covered the colloidal particles. It was then incubated in a water bath at 37°C for 3 h, and a trypsin solution (Enzyme:Sample = 1:50) was added. The solution was reacted at 37°C for 16 h to cleave the protein for producing a peptide.

The enzyme reaction was terminated after loading 50 μL 50% acetonitrile/5% trifluoroacetic acid (TFA) to lower the pH value. Ultrasonic vibration was used to extract the peptide. Subsequently, the extract was centrifuged, and its supernatant was transferred to a new microcentrifuge tube, then repeated twice, and the solution was drained in the microcentrifuge tube through a low‐temperature centrifugal concentrator. Then, a total of 10 μL 0.1% TFA was added to redissolve the peptide in a microcentrifuge tube and uses a C18 Zip‐Tip to sequentially absorb 10 μL 50% acetonitrile/0.1% TFA once and 10 μL 0.1% TFA; the steps were repeated three times. The microcentrifuge tube was repeatedly aspirated 10 times. The salt was washed out with 10 μL 0.1% TFA (this step was repeated 20 times). Then, the peptide was eluted to a new one with 10 μL 50% acetonitrile/0.1% formic acid (FA), and finally, a low‐temperature centrifugal concentrator to drain the solution in the microcentrifuge tube and stored at −20°C for further use.

#### 2.5.2 Liquid chromatography–tandem mass spectrometry analysis

A total of 40 μL of mobile phase (deionized water containing 1% Formic acid:ACN = 98:2) was added into the microcentrifuge tube to redissolve the extracted peptide sample, shook with ultrasonic waves for 1 min, and then centrifuged for 15 min at 15,000 *g*. The recovered supernatant was analyzed by liquid chromatography–tandem mass spectrometer (Orbitrap Elite™ Hybrid Ion Trap‐Orbitrap Mass Spectrometer). The column used was a C18 BEH column (Waters, Milford, MA) with a flow rate of 300 nL/min. The mobile phase comprised 1% formic acid (Buffer A) and acetonitrile (Buffer B). The gradient elution was performed within 60 min, increasing the concentration of mobile phase B from 5% to 35% (Buffer A: 1% FA in H_2_O, Buffer B: acetonitrile). The ion source was set with a voltage of 2.5 kV in positive mode at 90°C. Mass spectrometry scanning was performed in the m/z range of 350–1600, collecting ion signals in the charge states of 2+ to 6+.

#### 2.5.3 Tandem MS database comparison and peptides identification

The raw data obtained through liquid chromatography–tandem mass spectrometry (LC–MS/MS) were searched with the Swiss‐Prot database using the MS/MS ion search tool (http://www.matrixscience.com/cgi/search_form.pl?FORMVER=2&SEARCH=MIS). The search conditions and settings for the Mascot database were set as follows: trypsin for an enzyme, carbamidomethyl for fixed modifications, ± 10 ppm for peptide mass tolerance, oxidation for variable changes, and ± 0.6 Da for fragment mass tolerance. Monoisotopic was set to obtain the peptide.

#### 2.5.4 Bioactive peptides in silico analysis by BIOPEP database tools

Following the method described in Minkiewicz et al. (2019), the BIOPEP‐UWM database (https://biochemia.uwm.edu.pl/biopep‐uwm/) was utilized to predict potential active peptide types, quantities, physiological activities, and their sequence positions in the protein sequences. Multiple enzymes, including pancreatic elastase (EC 3.4.21.2), papain (EC 3.4.22.2), pepsin (EC 3.4.23.1), and bromelain (EC 3.4.22.32), were simulated for hydrolysis to analyze the active peptides generated from protein digestion by these enzymes.

### 2.6 In vitro analysis

#### 2.6.1 Enzymatic hydrolysis

After simulating enzymatic hydrolysis using the BIOPEP‐UWM database, the most suitable enzymes (papain, pepsin, bromelain) were selected based on the released bioactive peptides. To investigate the optimal enzyme‐to‐substrate ratio (E/S) and the optimal hydrolysis time, the methods of Lu et al. (2021) and Ozuna and León‐Galván (2017) were referenced and modified. Pumpkin seed protein powder was dissolved in ddH_2_O (3%, w/v), and the pH of the solution was adjusted to reach optimal enzyme activity conditions (Pepsin: 37°C, pH 2.0; Papain: 55°C, pH 7.0; Bromelain: 50°C, pH 8.0) using either 0.1 N HCl or 0.1 N NaOH. Enzymatic hydrolysis was performed at E/S ratios of 1%, 2%, and 3% (w/w) for 10 h. Every hour, 1 mL of hydrolysate was taken. To inactivate the enzymes, the hydrolysate was heated in a dry bath at 95°C for 15 min. The samples were then refrigerated at 4°C for further analysis. The remaining hydrolysates were freeze‐dried and stored at −20°C for future use.

#### 2.6.2 Quantitative analysis of soluble proteins

Pumpkin seed enzymatic hydrolysate was diluted with ddH_2_O to achieve a 10 mg/mL concentration. A total of 5 μL of the sample solution was dissolved with 25 μL of alkaline copper tartrate solution and 200 μL of Folin's reagent in a 96‐well plate. The mixture was subsequently reacted for 15 min in the dark and then measured by the absorbance using a microplate spectrophotometer at 750 nm. The protein content in the sample (mg/g) was determined by the BSA standard curve.

#### 2.6.3 Determination of peptide content

The peptide content was determined using the o‐phthaldialdehyde (OPA) method, according to Shori et al. (2022), with slight modifications. The peptide content in the hydrolysate was determined (mg/g) using the standard curve established with Gly‐Gly‐Gly as the standard sample.

#### 2.6.4 Fractionation

Fractionation was achieved by the ultrafiltration method reported by Famuwagun et al. (2020) with slight modifications. Briefly, different Molecular Weight Cut‐Off (MWCO) ultrafiltration membranes (10, 5, and 1 kDa) were rinsed with deionized water, shaken for 15 s (repeated twice), followed by the addition of 20% ethanol and shaking for 15 s (repeated twice), and then rinsed again with deionized water and shaken for 15 s (repeated twice) to clean the ultrafiltration membranes. Then, the ultrafiltration membranes were placed in an Amicon stirred ultrafiltration device, and 1 mg/mL concentration of pumpkin seed enzyme hydrolysate was added for separation based on different MW ranges. The obtained peptide fractions were classified into <1, 1–5, and 5–10 kDa. After freeze‐drying, the samples were stored at −20°C in the freezer. The yield of pumpkin seed enzyme hydrolysate was determined by the formulation below with W1 (g) as pumpkin seed enzyme hydrolysate powder and W2 (g) as resulting dry powder.

$$\begin{array}{cc} & \text{The yield of pumpkin seed enzyme hydrolysate after ultrafiltration}\;\left(\%\right)\\ & \;=W2/\text{W1}\times 100\%. \end{array}$$

#### 2.6.5 Angiotensin‐converting enzyme inhibitory activity

The in vitro angiotensin‐converting enzyme (ACE) inhibitory activity of pumpkin seed enzymatic hydrolysate was determined using the methods described by Aluko et al. (2015) with several modifications. A total 50 mM Tris–HCl buffer solution containing 0.3 M NaCl was dissolved with 0.5 mM N‐[3‐(2‐Furyl)acryloyl]‐L‐phenylalanyl‐glycyl‐glycine (FAPGG), and then the pH was adjusted to 7.5 by using 6 N HCl. Pumpkin seed enzymatic hydrolysate was mixed with a 50 mM Tris–HCl buffer (10 mg/mL concentration) to obtain the analytical sample solution. A total of 170 μL of 0.5 mM FAPGG was dissolved with 10 μL of ACE (0.5 U/mL, final activity of 25 mU) and 20 μL of the analytical sample solution for the assay, and the mixture was incubated for 30 min at 37°C in the dark. The absorbance at 345 nm (OD_345s_) was read using a microplate spectrophotometer. As a control group, 50 mM Tris–HCl buffer solution was used, and its absorbance was measured under the same conditions (OD_345c_).

The calculation formula is as follows:

$$\mathrm{ACE}\;\text{inhibitory}\;\left(\%\right)=\left[\frac{∆A_{\text{control}}-∆A_{\text{sample}}}{∆A_{\text{control}}}\right]\times 100\%$$

Δ*A*_sample_: OD_345 S0_ – OD_345 S30_ (The difference in absorbance values between the sample at 30 min and the initial absorbance value). Δ*A*_control_: OD_345 C0_ – OD_345 C30_ (The difference in absorbance values between the control group at 30 min and the initial absorbance value).

#### 2.6.6 Dipeptidyl peptidase‐IV inhibitory activity

According to the modified method reported by Kong et al. (2021), the in vitro DPP‐IV (Dipeptidyl peptidase‐IV) inhibitory activity of pumpkin seed enzymatic hydrolysate was measured. The pumpkin seed enzymatic hydrolysate was diluted at a concentration of 10 mg/mL in 100 mM Tris–HCl buffer (pH 8.0). A total 20 μL of the sample mixture was dissolved with 20 μL of Gly‐Pro‐p‐nitroanilide (1.6 mM) and incubated at 37°C for 10 min. Subsequently, 40 μL of DPP‐IV (8.0 U/L) was loaded to initiate the reaction, and the mixture was further incubated at 37°C for 60 min. Finally, a total of 80 μL of 1 M sodium acetate buffer (pH 4.0) was loaded to terminate the reaction, and the absorbance was recorded at 405 nm. The control group used 100 mM Tris–HCl buffer (pH 8.0) instead of the sample, and Diprotin A was used as a positive control reference for DPP‐IV inhibition. The calculation formula is as follows:

$$\mathrm{DPP}‐\mathrm{IV}\;\text{Inhibition}\;\left(\%\right)=\left[1-\frac{A_{405\left(\text{sample}\right)}-A_{405\left(\text{sample blank}\right)}}{A_{405\left(\text{positive control}\right)}-A_{405\left(\text{negative control}\right)}}\right]\times 100\%$$

*A*_405(sample)_: Absorbance value of the sample after the reaction. *A*_405(sample blank)_: Absorbance value of the reaction using Tris–HCl buffer (100 mM, pH 8.0) instead of DPP‐IV. *A*_405(positive control)_: Absorbance value of the reaction using Tris–HCl buffer (100 mM, pH 8.0) instead of the sample. *A*_405(negative control)_: Absorbance value of the reaction using Tris–HCl buffer (100 mM, pH 8.0) instead of the sample and DPP‐IV.

#### 2.6.7 Simulated gastrointestinal digestion

Based on the method described in Chait et al. (2020), simulated gastrointestinal digestion (SGID) test was conducted to simulate the action of human gastrointestinal enzymes. Pumpkin seed enzyme hydrolysate was mixed with ddH_2_O to obtain an analytical sample with a 10 mg/mL concentration. The solution pH was adjusted using 1 N HCl to 2.0 before adding pepsin (Enzyme/Substrate, E/S = 1:25, w/w). The mixture was reacted at 37°C for 2 h and then heated in a water bath for 10 min at 105°C to inactivate the enzymes. Next, the pH was adjusted to 7.0 using 1 N NaOH, and pancreatin (E/S = 1:25, w/w) was loaded. The reaction was conducted at 37°C for 4 h, followed by heating in a water bath for 10 min at 105°C to deactivate the enzymes. Subsequently, the samples were centrifuged, and the supernatant was recovered for freeze‐drying and stored in a −20°C refrigerator.

### 2.7 Statistical analysis

The data were analyzed by t‐tests or analysis of variance (ANOVA) along with Duncan's new multiple range test to estimate significant differences between the samples (*p* < .05).

## 3 RESULTS AND DISCUSSION

### 3.1 Protein content determination

Based on the preliminary experiment, the protein contents of pumpkin seeds after hot air drying (HAD) and freeze‐drying (FD) were 40.27% and 44.14%, respectively. This result indicates that pumpkin seeds are a good protein source. On the other hand, the protein contents of the HAD and FD groups of pumpkin seed protein isolates (PSPI) were 75.26% and 80.89%, respectively. It can be observed that PSPI obtained from the extraction process had a higher protein content.

### 3.2 Protein identification

**FIGURE 1:**
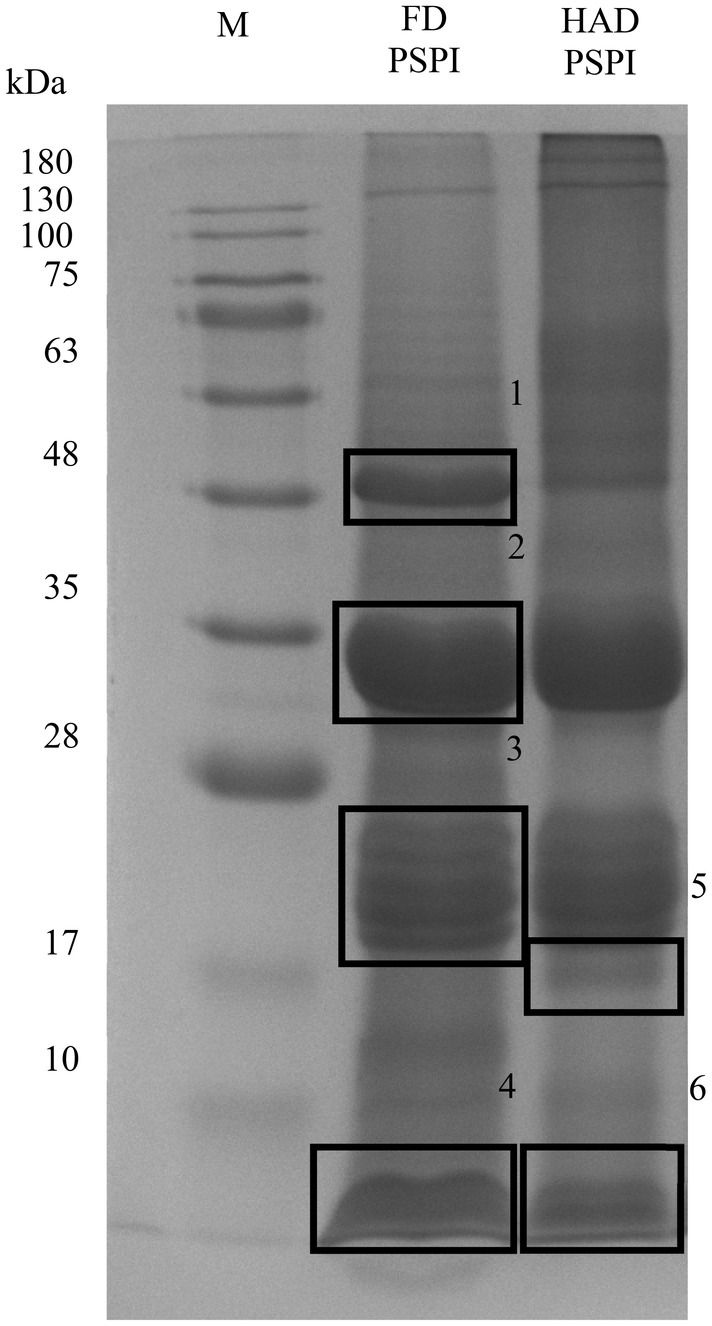
Sodium dodecyl sulfate‐polyacrylamide gel electrophoresis (SDS‐PAGE) of pumpkin seed protein isolate (PSPI) with 12% resolving gel. FD, Freeze‐drying; HAD, Hot air drying; M, Marker.

PSPI was subjected to SDS‐PAGE to observe and separate the different MW distributions of the protein. The presence of six protein bands in PSPI with MW distributed around 48, 35, 17–28 kDa (Figure 1). According to Rezig et al. (2013), protein bands with higher MW (50 and 36 kDa) were attributed as 11S protein, and lower MW (14–24 kDa) corresponds to 2S albumin fragments.

**FIGURE 2:**
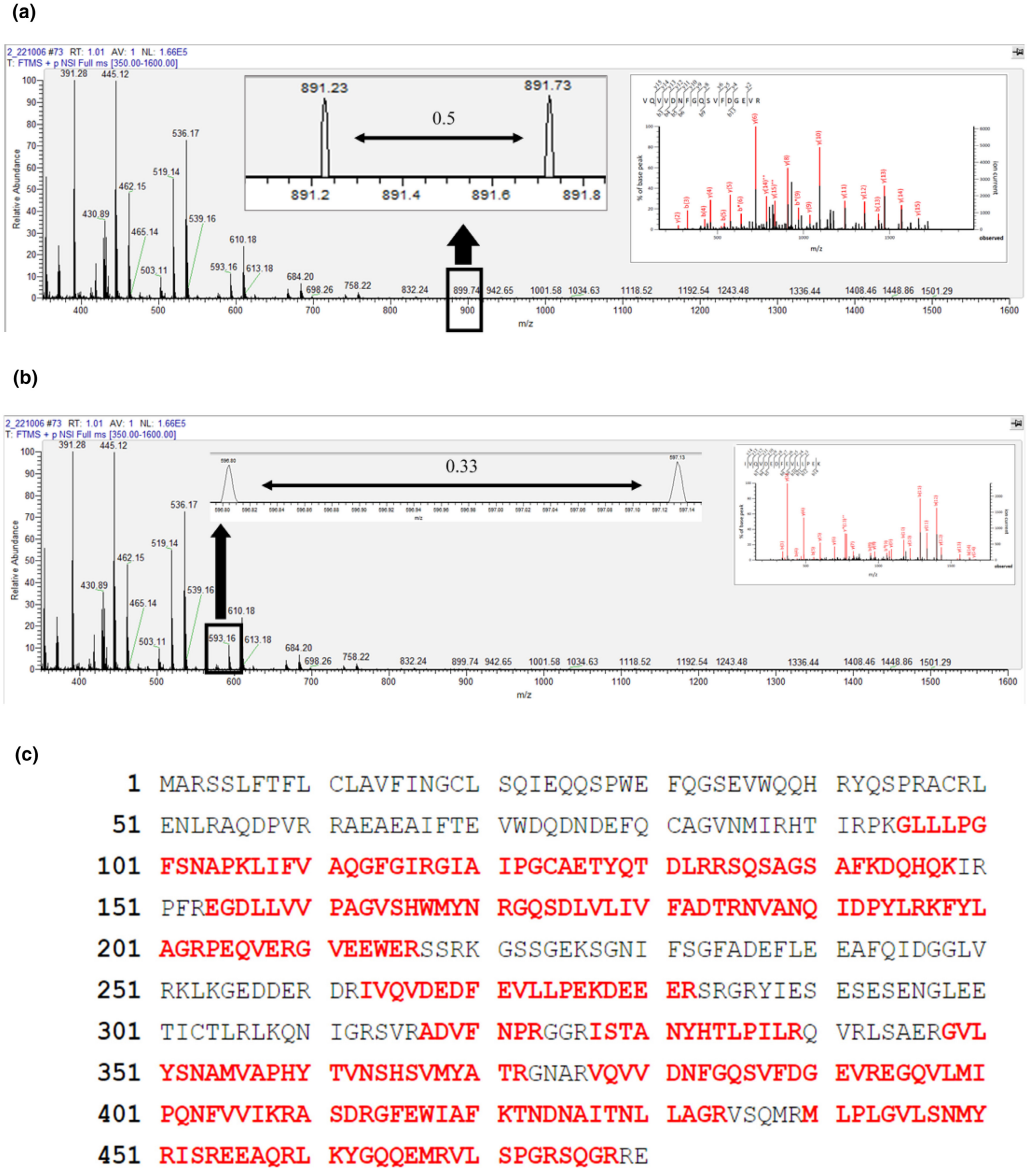
(a, b) Examples of LC–MS/MS spectra of a doubly charged peptide (VQVVDNFGQSVFDGEVR, MW: 947.96, 2^+^) and a triply charge peptide from pumpkin seed protein isolate (SDS‐PAGE band 2); (c) Sequence of identified tryptic peptides from pumpkin seed protein isolate (PSPI) (red letters) matched the 11S globulin subunit beta (Swiss‐Prot accession number: P13744).

Taking PSPI band 2 as an example, the first‐stage mass spectrum (MS^1^) attained after the initial collision in the mass spectrometer is shown in Figure 2. Due to the presence of isotopes, peptides with the same sequence differ by one mass unit. Therefore, peptides with 2 and 3 charges will exhibit adjacent peaks in the mass spectrum with m/z values (Nichols & White, 2009) differing by 0.5 (Figure 2a) and 0.33 (Figure 2b). According to the LC‐MS/MS and Mascot database analysis results, most of the peptides generated after in‐gel digestion carry double or triple charges. Two peaks had m/z differences of 0.5 and 0.33. During collision‐induced dissociation (CID), inert gases such as nitrogen or helium collide with selected precursor ions, generating ion fragments to infer protein sequences. The MS^2^ spectrum of the precursor ion with a stronger signal (m/z 947.96) is shown in Figure 2a. The *b* and *y* peaks in the spectrum are naming conventions for ion fragments, representing the ions produced by cleavages at the N‐ and C‐termini, respectively (Giese et al., 2016). The numbers in subscript indicate the amino acids quantity in the fragment ion and their position within the peptide. By comparing and calculating against the database, the tryptic peptide sequence corresponding to m/z 947.96 is determined to be VQVVDNFGQSVFDGEVR. Figure 2c illustrates the sequence of identified tryptic peptides from pumpkin seed protein isolate (PSPI) that matched the 11S globulin subunit beta (Swiss‐Prot accession number: P13744). The identified proteins from PSPI have been compiled in Table 1. Based on their sequence coverage and protein scores, three main proteins present in PSPI have been selected: 11S globulin subunit beta, 2S seed storage albumin protein, and chaperonin CPN60‐1 (mitochondrial). Among these, 11S globulin subunit beta has the highest sequence coverage, ranging from 35% to 58%, followed by 2S seed storage albumin protein (12% to 29%), and finally, chaperonin CPN60‐1 (8% to 20%). The protein scores for these proteins are 663–6109, 75–231, and 118–390, respectively. Their MWs are approximately 54.9, 17.0, and 61.4 kDa. 11S globulin subunit beta and 2S seed storage albumin are vital to plant seed storage proteins. Their molecular structures are compact and resistant to degradation in the human digestive system, making them suitable for developing functional food ingredients (Tan‐Wilson & Wilson, 2012). Therefore, the next step will be to use BIOPEP‐UWM to simulate enzyme hydrolysis and predict potential bioactive peptides for these three main proteins found in pumpkin seeds.

**TABLE 1:** The identified proteins from the pumpkin seed protein isolate (PSPI) and their characteristics in Mascot database.

Band	Protein name	Organism	Accession number	Score	Sequence coverage (%)	Molecular weight from database (kDa)
1	11S globulin subunit beta	*Cucurbita maxima*	11SB_CUCMA	994	51	54.9
	Citrate synthase	*Cucurbita maxima*	CYSZ_CUCMA	418	26	56.8
	Chaperonin CPN60‐1, mitochondrial	*Cucurbita maxima*	CH62_CUCMA	390	28	61.4
	Phosphoglycerate kinase 1, chloroplastic	*Arabidopsis thaliana*	PGKH1_ARATH	212	17	50.1
	2S seed storage albumin protein	*Cucurbita maxima*	2SS_CUCMA	86	12	17.0
2	11S globulin subunit beta	*Cucurbita maxima*	11SB_CUCMA	2447	54	54.9
	Isocitrate lyase	*Cucurbita maxima*	ACEA_CUCMA	203	16	64.7
	Chaperonin CPN60‐1, mitochondrial	*Cucurbita maxima*	CH61_CUCMA	118	9	61.3
	2S seed storage albumin protein	*Cucurbita maxima*	2SS_CUCMA	84	23	17.0
	40S ribosomal protein S3a	*Daucus carota*	RS3A_DAUCA	271	18	29.9
	Phosphoglycerate kinase 1, chloroplastic	*Arabidopsis thaliana*	PGKH1_ARATH	291	12	50.1
3	11S globulin subunit beta	*Cucurbita maxima*	11SB_CUCMA	6109	58	54.9
	40S ribosomal protein S9‐1	*Arabidopsis thaliana*	RS91_ARATH	127	21	23.0
	Translationally controlled tumor protein homolog	*Cucurbita maxima*	TCTP_CUCMA	170	21	19.1
	11S globulin seed storage protein 1	*Carya illinoinensis*	11S1_CARIL	166	6	58.3
	2S seed storage albumin protein	*Cucurbita maxima*	2SS_CUCMA	75	13	17.0
4	11S globulin subunit beta	*Cucurbita maxima*	11SB_CUCMA	930	49	54.9
	2S seed storage albumin protein	*Cucurbita maxima*	2SS_CUCMA	210	30	17.0
	Translationally controlled tumor protein homolog	*Cucurbita maxima*	TCTP_CUCMA	107	15	19.1
	Inhibitor of trypsin and hageman factor	*Cucurbita maxima*	ITH5_CUCMA	68	29	74.7
5	11S globulin subunit beta	*Cucurbita maxima*	11SB_CUCMA	4027	48	54.9
	11S globulin seed storage protein 1	*Carya illinoinensis*	11S1_CARIL	153	6	58.3
	2S seed storage albumin protein	*Cucurbita maxima*	2SS_CUCMA	150	12	17.0
	18.1 kDa class I heat shock protein (fragment)	*Medicago sativa*	HSP11_MEDSA	134	21	16.4
6	11S globulin subunit beta	*Cucurbita maxima*	11SB_CUCMA	663	35	54.9
	2S seed storage albumin protein	*Cucurbita maxima*	2SS_CUCMA	231	26	17.0
	Rubber elongation factor protein	*Hevea brasiliensis*	REF_HEVBR	64	2	14.7
	11S globulin	*Juglans nigra*	JUGN4_JUGNI	52	4	58.4

### 3.3 In silico analysis by BIOPEP‐UWM

Through protein separation, digestion, mass spectrometry analysis, and database matching, the main proteins in pumpkin seeds were identified as 11S globulin subunit beta, 2S seed storage albumin, and chaperonin CPN60‐1. Using BIOPEP‐UWM, Table 2 summarized the results of the analysis of potential bioactive peptides from 11S globulin subunit beta, 2S seed storage albumin, and chaperonin CPN60‐1. The frequency of bioactive peptides refers to the proportion of specific bioactive peptides among the total number (Ribeiro et al., 2021). According to their quantities and frequencies, all three proteins show the highest abundance of DPP‐IV inhibitory peptides, followed by ACE inhibitory peptides. It can be observed that the bioactive peptides consist of oligopeptides composed of 2 to 6 amino acids with ACE inhibitory, antioxidant, and DPP‐IV inhibitory activities. Some peptide segments, such as VW, IR, and MY, exhibit multiple activities simultaneously.

**TABLE 2:** Number of potential bioactive peptides from pumpkin seed protein isolate (PSPI) using BIOPEP‐UWM tool.

Protein	ACE inhibitor	DPP‐IV inhibitor	Other activity
11S globulin subunit beta	112 (0.32)	144 (0.41)	72 (0.21)
2S seed storage albumin protein	39 (0.28)	55 (0.40)	38 (0.27)
Chaperonin CPN60‐1, mitochondrial	97 (0.31)	136 (0.44)	64 (0.20)

*Note*: Values provided in bracket are the frequency of peptide.

Abbreviations: ACE, Angiotensin‐converting enzyme; DPP‐IV, Dipeptidyl peptidase‐IV.

The simulation of enzyme hydrolysis using several common enzymes through the BIOPEP‐UWM database tool (Table 3) showed that papain, bromelain, and pepsin generate a relatively more significant number of bioactive peptides from 11S globulin subunit beta, 2S seed storage albumin, and chaperonin CPN60‐1. Their production costs are relatively low and inexpensive compared to other complex enzymes, making them suitable for large‐scale industrial applications (Ashaolu & Yupanqui, 2018).

**TABLE 3:** Number of predicted bioactive peptides to be released from 11S globulin subunit beta, 2S seed storage albumin protein and Chaperonin CPN60‐1, mitochondrial using BIOPEP's enzyme tool.

Protease	11S globulin subunit beta			2S seed storage albumin protein			Chaperonin CPN60‐1, mitochondrial		
	ACE	AO	DPP‐IV	ACE	AO	DPP‐IV	ACE	AO	DPP‐IV
Chymotrypsin A	14	3	13	2	2	ND	5	2	6
Chymotrypsin C	19	5	27	8	2	8	10	ND	11
Pancreatic	26	4	49	4	1	7	43	3	51
Papain	31	3	45	5	2	14	34	2	61
Pepsin	32	2	44	12	3	11	46	1	59
Proteinase K	20	2	26	1	1	2	10	2	23
Stem bromelain	29	2	36	7	1	9	47	4	45
Thermolysin	18	1	17	ND	ND	5	22	2	27
Trypsin	4	5	3	1	ND	1	7	ND	3

Abbreviations: ACE, Angiotensin‐converting enzyme inhibitor; AO, Antioxidative; DPP‐IV, Dipeptidyl peptidase‐IV inhibitor; ND, No data.

### 3.4 In vitro hydrolysis of PSPI

**FIGURE 3:**
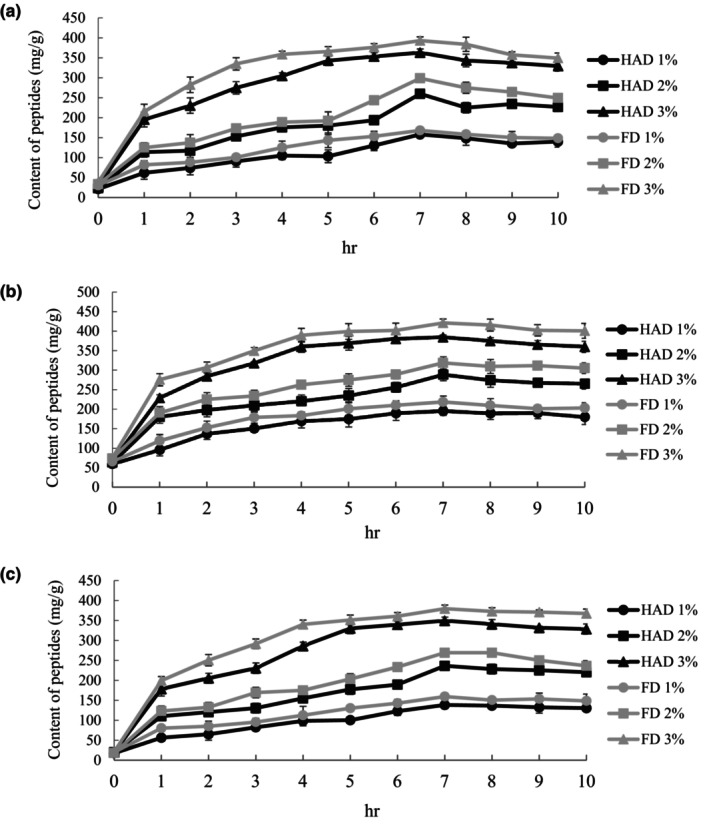
Peptide content of (a) papain, (b) pepsin, (c) bromelain hydrolysates with different enzymes/substrates (E/S) at 1–10 h. HAD: Hot air drying, FD: Freeze‐drying. All data are shown as mean ± SD (*n* = 3).

Bioactive peptides are specific peptide sequences within proteins released through hydrolysis from the parent protein and can interact with specific targets and modulate physiological functions. In the initial stages of hydrolysis (0–4 h), all groups showed a rapid increase in peptide content by the extensive peptide bond breakdown during the early stages of hydrolysis, leading to peptide content rapid increase (Damrongsakkul et al., 2008). The current study showed that the hydrolysis peaked at 7 h, followed by a more gradual increase as hydrolysis time increased (Figure 3). Both HAD and FD groups had the highest peptide content at an enzyme/substrate ratio of 3%. Specifically, the HAD group had papain hydrolysate at 363.20 mg/g, pepsin hydrolysate at 383.90 mg/g, and bromelain hydrolysate at 349.48 mg/g peptide contents. On the other hand, the FD group had papain hydrolysate at 398.26 mg/g, pepsin hydrolysate at 420.83 mg/g, and bromelain hydrolysate at 375.26 mg/g peptide contents. High drying temperature used in the HAD group may have caused the degradation of some amino acids such as phenylalanine, leucine, isoleucine, and tyrosine, leading to partial peptide degradation and reducing the peptide content in the hydrolysate. In contrast, most peptide structures were preserved and not degraded in the FD group, resulting in higher peptide content (Gan et al., 2020). Additionally, pepsin has a solid ability to cleave short‐chain peptides, thereby releasing more peptides (Ge et al., 2022). The optimal hydrolysis conditions were chosen based on the results as a 3% enzyme/substrate ratio and a hydrolysis time of 7 h.

**FIGURE 4:**
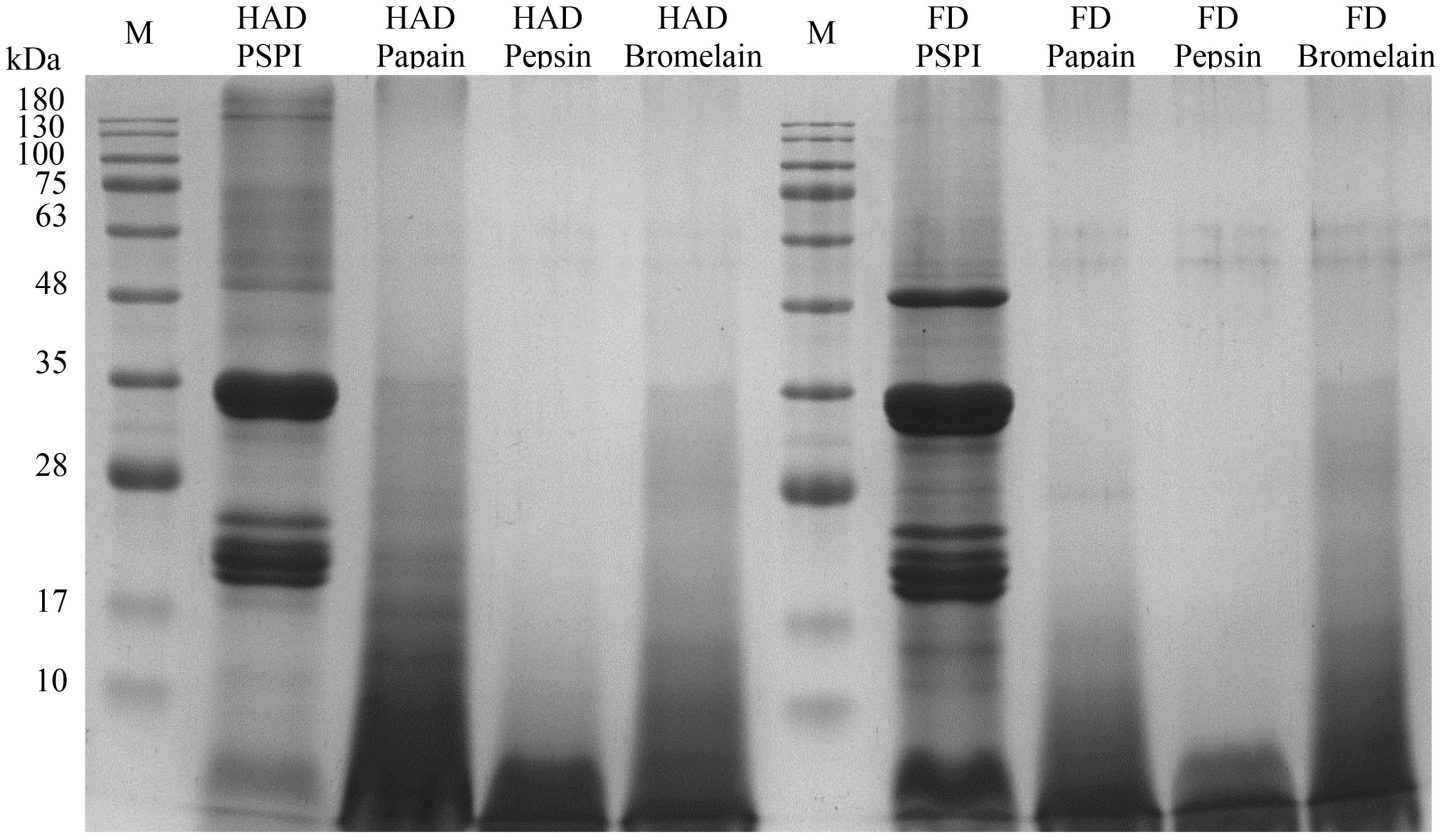
SDS‐PAGE of PSPI and its enzyme hydrolysates with 12% resolving gel. M: Marker, HAD: Hot air drying, FD: Freeze‐drying, Papain: Papain hydrolysate, Pepsin: Pepsin hydrolysate, Bromelain: Bromelain hydrolysate.

The soluble protein content and yield of pumpkin seed enzyme hydrolysates are presented in Table 4. The yield of pumpkin seed enzyme hydrolysates ranged from 68.76% to 83.38%, with pepsin hydrolysate having the highest yield (79.46% to 83.38%). The broad cleavage specificity of pepsin enables the generation of various peptide fragments (Awosika & Aluko, 2019). From the results shown in Figure 4, it can be observed that the MW distribution was around 48, 35, and 17–28 kDa, mostly shifting to below 17 kDa, significantly lower than the pre‐hydrolysis distribution.

**TABLE 4:** Soluble protein content and yield of pumpkin seed hydrolysate.

Hydrolysates	Drying method	Protein contents (%)	Yield (%)
Papain	HAD	34.59 ± 1.03^b^	70.16 ± 0.19^b^
	FD	38.03 ± 0.63^a^	78.50 ± 0.11^a^
Pepsin	HAD	45.07 ± 0.33^b^	79.46 ± 0.26^b^
	FD	47.07 ± 0.49^a^	83.38 ± 0.21^a^
Bromelain	HAD	40.13 ± 0.60^b^	72.61 ± 0.19^a^
	FD	42.88 ± 0.33^a^	68.76 ± 0.10^b^

*Note*: Different letters represent significant differences in the same column (*p* < .05). All data are shown as mean ± SD (*n* = 3).

Abbreviations: FD, Freeze‐drying; HAD, Hot air drying.

### 3.5 Bioactive activity of enzyme hydrolysates

**FIGURE 5:**
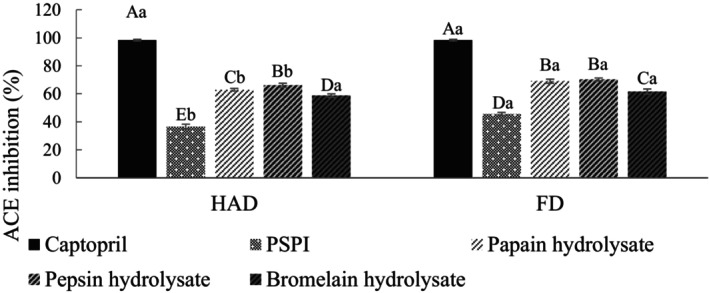
ACE inhibition activity of PSPI and its hydrolysates. FD, Freeze‐drying; HAD, Hot air drying; PSPI, Pumpkin seed protein isolate. Captopril was used as a reference inhibitor. ACE inhibition activity was tested at a final concentration of 10 mg/mL. All data are shown as mean ± SD (*n* = 3). Different uppercase letters represent significant differences between the same samples at each drying method (*p* < .05), and different lowercase letters represent significant differences in the same drying method (*p* < .05).

ACE participates in the renin–angiotensin system and plays a crucial role in converting angiotensin‐I into angiotensin‐II, which leads to vasoconstriction, aldosterone synthesis, and the inhibition of bradykinin activity. ACE inhibitors lower blood pressure (Morato et al., 2017). High ACE inhibitory activity peptides often possess hydrophobic and positively charged amino acids such as arginine and lysine, or they have a combination of aliphatic (e.g., isoleucine, glycine, leucine) and aromatic amino acids at the N‐terminal and C‐terminal regions (Wang et al., 2020). These characteristics allow the peptides to interact with the ACE structure, leading to inhibition. Figure 5 shows the ACE inhibitory activity of PSPI and its hydrolysates. The ACE inhibition of PSPIs was 36.59% (HAD) and 45.63% (FD). The results indicate that the ACE inhibitory activity increases after enzymatic hydrolysis, with the pepsin hydrolysate showing the highest activity at 70.26. Pepsin, an endopeptidase with broad specificity, can cut peptide bonds among aromatic and hydrophobic amino acids (Fruton, 1970), producing higher ACE inhibitory activity peptides.

**FIGURE 6:**
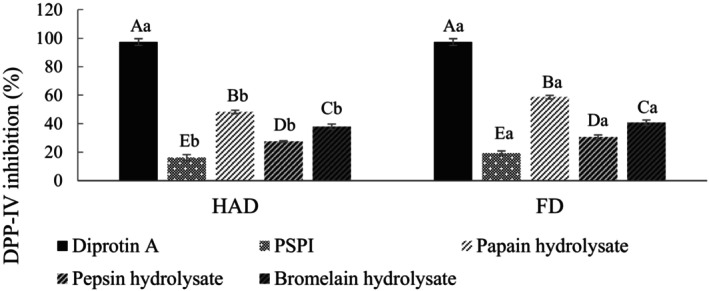
DPP‐IV inhibition activity of PSPI and its hydrolysates. FD, Freeze‐drying; HAD, Hot air drying; PSPI, Pumpkin seed protein isolate. Diprotin A (Ile‐Pro‐Ile) was used as a reference inhibitor. DPP‐IV inhibitory activity was tested at a final concentration of 10 mg/mL. All data are shown as mean ± SD (*n* = 3). Different uppercase letters represent significant differences between the same samples at each drying method (*p* < .05), and different lowercase letters represent significant differences in the same drying method (*p* < .05).

Dipeptidyl peptidase‐IV (DPP‐IV) is a metabolic enzyme that degenerates GLP‐1 and GIP in various organs, preventing normal insulin secretion and causing an imbalance in blood glucose concentration. The DPP‐IV inhibitory activities of PSPI and its hydrolysate are shown in Figure 6. Pre‐hydrolysis activities of PSPI were HAD: 16.20% and FD: 19.31%. After hydrolysis, the inhibitory activities significantly increased, ranging between 27.58% and 58.62%. Among them, the highest inhibitory activity was revealed by the FD group's papain hydrolysate, which affected the DPP‐IV inhibitory activity of the hydrolysate and was influenced by the proline content in peptides, where peptides with higher proline content show better DPP‐IV inhibition (Roghayeh et al., 2022). Compared to freeze‐drying, hot air drying may reduce the hydrolysate's proline content (Kiettiolarn et al., 2022).

### 3.6 Simulated gastrointestinal digestion of enzyme hydrolysates

**FIGURE 7:**
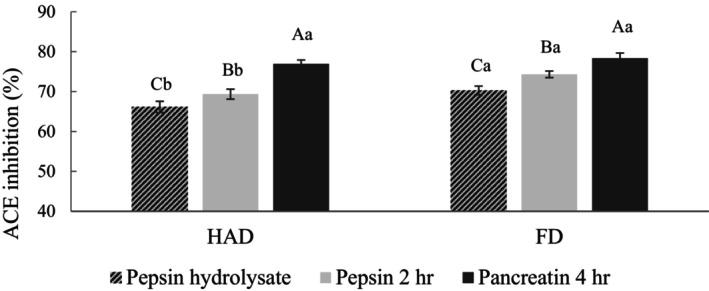
ACE inhibition activity of pumpkin seed pepsin hydrolysate after simulated gastrointestinal digestion. FD, Freeze‐drying; HAD, Hot air drying. ACE inhibition activity was tested at a final concentration of 10 mg/mL. All data are shown as mean ± SD (*n* = 3). Different uppercase letters represent significant differences between the same samples at each drying method (*p* < .05), and different lowercase letters represent significant differences in the same drying method (*p* < .05).

The pepsin hydrolysate revealed the highest antioxidant activity among the three pumpkin seed enzyme hydrolysates. Therefore, it was chosen as the sample for simulated gastrointestinal digestion (SGID) and bioactive activity experiments. SGID is a commonly used method to investigate bioactive peptide stability. Since the pepsin hydrolysate showed the best ACE inhibitory activity, it was chosen as the sample for subsequent experiments. Figure 7 shows that the ACE inhibitory activity of the pepsin hydrolysate significantly increased after SGID. The ACE inhibitory activity of the HAD and FD groups increased from 62.93% and 69.06% to 76.93% and 78.34%. Previous studies have confirmed that ACE inhibitory peptides are primarily low MW peptide segments, and small peptides are less vulnerable to the influence of digestive enzymes and pH, maintaining their high activity and structural integrity in the gastrointestinal digestive fluids (Li et al., 2018). Additionally, pancreatin can specifically cleave peptide bonds adjacent to positively charged amino acids lysine and arginine, thereby enhancing ACE inhibitory activity (Liu et al., 2020). The ACE inhibitory activities for the HAD and FD groups were 81.49% and 83.27%, with no significant difference.

**FIGURE 8:**
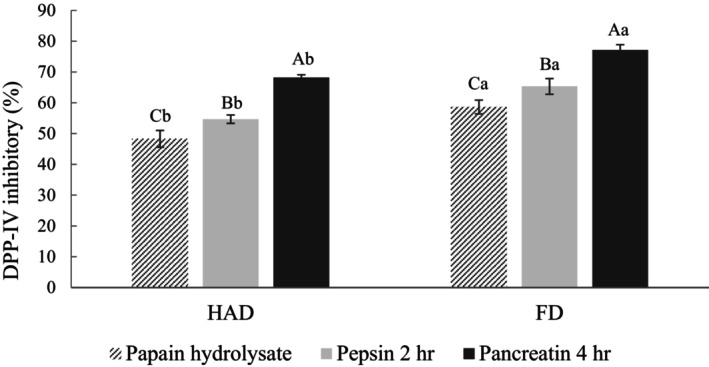
DPP‐IV inhibition activity of pumpkin seed papain hydrolysate after simulated gastrointestinal digestion. FD, Freeze‐drying; HAD, Hot air drying. DPP‐IV inhibition activity was tested at a final concentration of 10 mg/mL. All data are shown as mean ± SD (*n* = 3). Different uppercase letters represent significant differences between the same samples at each drying method (*p* < .05), and different lowercase letters represent significant differences in the same drying method (*p* < .05).

The papain hydrolysate was chosen for subsequent experiments since exhibited the highest DPP‐IV inhibitory activity. Figure 8 shows the DPP‐IV inhibitory activity of the papain hydrolysate after SGID. The DPP‐IV inhibitory activity of the HAD and FD groups increased from 48.27% and 58.62% to 68.19% and 77.13%, attributed to the release of more peptide segments related to DPP‐IV inhibitory activity by gastrointestinal enzymes (Kong et al., 2021). The results suggest that DPP‐IV inhibitory peptides possess good gastrointestinal digestion stability, as their activity does not decrease due to excessive enzymatic hydrolysis.

### 3.7 Bioactive activity of peptides

Ultrafiltration (UF) can eliminate insoluble and large molecular‐weight proteins and peptides, producing smaller molecular‐weight peptide fragments (Fan et al., 2012). The bioactive peptides' amino acid composition and sequence can affect their activity, with smaller peptides typically exhibiting higher physiological activity (Cotabarren et al., 2019). To investigate whether the MW influences the physiological activity of the pumpkin seed enzyme hydrolysate, ultrafiltration was conducted using UF membranes with MWCO of 10, 5, and 1 kDa. This process separated the hydrolysate into three fractions (<1, 1–5, 5–10 kDa), and their physiological activity was further determined.

**FIGURE 9:**
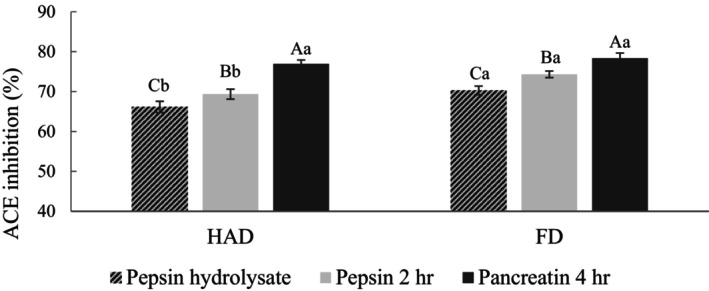
ACE inhibition activity of pumpkin seed pepsin hydrolysate after simulated gastrointestinal digestion. FD, Freeze‐drying; HAD, Hot air drying. ACE inhibition activity was tested at a final concentration of 10 mg/mL. All data are shown as mean ± SD (*n* = 3). Different uppercase letters represent significant differences between the same samples at each drying method (*p* < .05), and different lowercase letters represent significant differences in the same drying method (*p* < .05).

The ACE inhibitory activity of the pepsin hydrolysate after ultrafiltration is shown in Figure 9. The results indicate that the separated peptide segments have higher inhibitory activity, with the <1 kDa fraction showing the highest activity. The ACE inhibitory activities for the HAD and FD groups were 81.49% and 83.27%, with no significant difference. This is because lower molecular weight ACE inhibitory peptide segments are more likely to cleave the ACE active site and inhibit its interaction, resulting in higher inhibitory activity (Paul et al., 2021). The results suggest that ultrafiltration contributes to generating low MW peptides and enhances ACE inhibitory activity.

**FIGURE 10:**
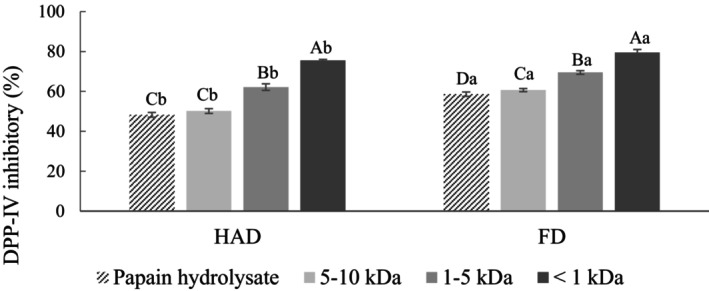
DPP‐IV inhibition activity of pumpkin seed papain hydrolysate after ultrafiltration. FD, Freeze‐drying; HAD, Hot air drying. DPP‐IV inhibitory activity was tested at a final concentration of 10 mg/mL. All data is shown as mean ± SD (*n* = 3). Different uppercase letters represent significant differences between the same samples at each drying method (*p* < .05), and different lowercase letters represent significant differences in the same drying method (*p* < .05).

The results of DPP‐IV inhibitory activity of the papain hydrolysate after ultrafiltration are shown in Figure 10. It can be observed that as the MW of the peptide segments decreased, their inhibitory activity increased, with the <1 kDa fraction exhibiting the highest DPP‐IV inhibitory activity. The DPP‐IV inhibitory activities for the HAD and FD groups were 75.57% and 79.55%, respectively. The result suggested that the increase in number of lower MW peptides after ultrafiltration makes it easier for them to enter and bind to the active site of DPP‐IV, leading to competitive inhibition and an increase in DPP‐IV inhibitory activity (Connolly et al., 2015; You et al., 2022). The results suggest that ultrafiltration generates low MW peptides and enhances DPP‐IV inhibitory activity.

## 4 CONCLUSION

Protein isolate prepared from hot air‐dried and freeze‐dried pumpkin seed exhibited in vitro inhibition activities, including ACE and DPP‐IV inhibitions. Based on the analysis using the BIOPEP‐UWM bioactive peptide database, enzymatic hydrolysis of pumpkin seeds by papain, pepsin, and bromelain may produce a higher yield of peptides and generate peptides with ACE inhibitory and DPP‐IV inhibitory activities. The hydrolysates were divided into hot air drying (HAD) and freeze‐drying (FD) groups based on the drying method. The results revealed that pepsin hydrolysate from the FD group exhibited the highest ACE inhibitory activities of 70.26%. The FD group's DPP‐IV inhibitory activity of papain hydrolysate showed the highest value (58.62%). Further investigations through simulated gastrointestinal digestion and ultrafiltration revealed that pepsin and pancreatin digest, and the <1 kDa peptide fraction obtained by ultrafiltration exhibited higher activity. This indicated that the bioactive peptides from pumpkin seeds have good stability in the gastrointestinal tract, and smaller peptide fragments contribute to higher bioactivity. The results suggested that freeze‐drying preserves the peptide structures and functional characteristics better. However, the hydrolysates obtained from hot air drying also released various active peptides, with lower costs and shorter processing time, making them suitable for large‐scale industrial production. Further evaluation of PSPI activity and effects can be conducted using cell or animal experiments, which can be utilized as functional foods.

## AUTHOR CONTRIBUTIONS

**Chu‐Ti Lin:** Data curation (equal); formal analysis (equal); investigation (equal); methodology (equal); visualization (equal); writing – original draft (lead). **Lhumen A. Tejano:** Writing – review and editing (equal). **Fenny Crista A. Panjaitan:** Writing – review and editing (equal). **Vinny Nabila Surya Permata:** Writing – review and editing (equal). **Tesalonika Sevi:** Writing – review and editing (equal). **Yu‐Wei Chang:** Conceptualization (lead); funding acquisition (lead); methodology (equal); resources (lead); supervision (lead); validation (lead); visualization (equal); writing – review and editing (equal).

## CONFLICT OF INTEREST STATEMENT

There are no conflicts of interest.

## ETHICS STATEMENT

This report does not conduct any human or animal tests.

## Data Availability

The data that support the findings of this study are available from the corresponding author upon reasonable request.
